# Effects of Intravenous Lipid Emulsion on Tramadol-Induced Seizure; a Randomized Clinical Trial

**DOI:** 10.22037/aaem.v9i1.1070

**Published:** 2021-02-20

**Authors:** Amir Mohammad Kazemifar, Zohreh Yazdi, Abbas Bedram, Javad Mahmoudi, Mojtaba Ziaee

**Affiliations:** 1 Faculty of Medicine, Qazvin University of Medical Sciences, Qazvin, Iran.; 2 Neuroscience Research Center, Tabriz University of Medical Sciences, Tabriz, Iran.; 3 Medicinal Plants Research Center, Maragheh University of Medical Sciences, Maragheh, Iran.

**Keywords:** Tramadol, soybean oil, phospholipid emulsion, Poisoning, Seizure, Clinical trial

## Abstract

**Introduction:**

There are numerous studies on the efficacy of intralipid emulsion (ILE) in various xenobiotic toxicities. This study aimed to evaluate the potential role of ILE as an antidote in tramadol-induced seizure.

**Methods:**

A single-blind clinical trial was undertaken to establish the efficacy and safety of ILE in patients with acute tramadol intoxication, who referred to Booali Hospital in Qazvin. Patients were randomly assigned to 2 groups. The Control group received standard care while the intervention group received intralipid emulsion (ILE) 20% in addition to the standard care. The occurrence of in-hospital seizure was compared between the groups.

**Results:**

80 patients who abused tramadol and met the study criteria were randomly assigned to either the intervention (40 cases) or the control (40 cases) group. Seizure occurred in 44 (56%) patients before admission to the emergency department. There were not any statistical differences between the groups regarding sex distribution (p=0.513) and mean age (p=0.19), presenting vital signs (p < 0.05), laboratory findings (p < 0.05), and mean abused dose of tramadol (p = 0.472) as well as occurrence of prehospital seizure (p = 0.7). In-hospital seizure occurred in 15 (18.75%) cases (all in the control group; p < 0.001). The mean duration of admission was 2.01 ± 1.13 days in the control group and 2.15 ± 1.04 days in the intervention group (p = 0.6). The number needed to treat for ILE to prevent tramadol-induced seizure was 2.7 (37.5% absolute risk reduction).

**Conclusions:**

The findings of this study supported ILE administration, as an adjunct to standard antidote protocols, in tramadol intoxication to prevent tramadol-induced seizures.

## Introduction

Tramadol is a synthetic analgesic drug that exerts opioid and non-opioid effects, acting predominantly on the central nervous system (CNS). It is prescribed to treat moderate to severe pain such as postoperative or chronic pain (1). Although it appears to be a safe and effective analgesic, there are evidence that have linked tramadol to illegal abuse and poisoning and even anaphylactoid reactions (2). The prevalence of tramadol abuse and poisoning has risen significantly in the Middle East and many other countries (3). Major clinical findings of tramadol intoxication are seizures, apnea, hemodynamic changes (bradycardia or tachycardia and hypotension), and coma (4). Tramadol-induced seizures are mostly not associated with dose and occur within the first 4-6 hours (5). 

Several studies have reported successful resuscitation of these patients through the utilization of intravenous lipid emulsion (ILE) as an antidote for the treatment of hemodynamic or neurological symptoms of poisoning with lipophilic xenobiotics (6). Although several mechanisms have been postulated for the resuscitative properties of ILE, two theories are more interesting: Lipid sink (partitioning) and enhanced metabolism theories. The partitioning theory suggests that ILE administration compartmentalizes the lipophilic drugs into the lipid phase and detaches them from the target tissues. Decreased drug concentrations in blood flow facilitate the elimination of the xenobiotics from the affected organs through the generation of a concentration gradient (7).

The enhanced metabolism theory argues that infusion of ILE elevates cardiac metabolism and has inotropic effects. Recent studies claimed that ILE administration increased left ventricular contractility. Both hypotheses were established based on pieces of evidence indicating that lipid therapy shows efficacy on a variety of xenobiotic groups with different receptor specificities. Thus, lipid therapy may counteract particular poisons either through receptor-independent pathways or by a common downstream mechanism.

Despite the existence of numerous studies on ILE efficacy and the satisfactory findings in various xenobiotics toxicities, limited clinical researches have studied ILE in patients who have overdosed on tramadol and experienced seizures (8). This study aimed to evaluate the potential role of ILE as an antidote in tramadol-induced seizure. 

## Methods

**Study design and setting**

This randomized, single-blinded, clinical trial was conducted on 80 patients with acute tramadol poisoning who were referred to the emergency department (ED) of Booali Hospital, Qazvin, Iran, between August 2017 and September 2018. The research procedure was approved by the Ethics Committee of Qazvin University of Medical Science (Code: IR.QUMS.REC.1395.267) and was registered at the Iranian Registry of Clinical Trials under registration number IRCT2017050120951N3. Written informed consent was received from all patients after a brief presentation, and before they participated in the study. The research was designed as a pilot and thus, had a relatively limited sample size.

**Study population**

80 consecutive cases, who were admitted to the ED during the study period, were studied. Patients were eligible for participation in the study if they met the following criteria: 1) Adults (16 years and above), 2) tramadol ingestion (based on patient’s history), 3) clinical presentations of tramadol overdose and 4) referring to ED within 4 hours of tramadol intake.

Patients were excluded from the study if they met the following conditions: 1) multiple drug ingestion, 2) underlying heart disease, 3) hypersensitivity to the drug, 4) any previous history of seizure, and 5) abnormality in blood oxygen, electrolyte, and biochemical analysis. Multiple drug intakes were characterized based on history with or without confirmed urine or serum drug screening test.

**Data gathering**

The baseline characteristics of the patients including sex, age, vital signs (pulse rate, respiratory rate, blood pressure, Glasgow coma scale, and oxygen saturation), history of seizure before admission to ED, dosage of abused drug, and lab tests were evaluated on admission of patients. The patients were randomly assigned to control and intervention groups using a computer-generated randomization schedule/table and simple randomization method. All participants were blinded to the type of treatment received until the completion of the study. Blood samples were drawn for assessing blood sugar, blood urea nitrogen (BUN), creatinine (Cr), sodium (Na^+^), potassium (K^+^), liver function, hematological parameters. The diagnosis of seizures before admission to the emergency department was done based on an accurate medical history and reports taken from the witnesses who accompanied the patient and verified via scars of tongue biting. In-hospital events such as seizures were accurately monitored by the well-trained medical staff. An internal medicine specialist was responsible for data gathering.

***Intervention***

The patients in the intervention group received a single dose of 12 mL/kg of intralipid emulsion (ILE) 20% (30% as a bolus dose and the remaining infused over 3 hours) after stabilization along with routine treatment, while the control group only received the standard medical treatment including gut decontamination and adequate supportive care. The patients were monitored for vital parameters and seizures every 10 minutes.

**Outcomes**

The main outcome of present study was the effect of ILE on occurrence and frequency of tramadol-induced seizure.

**Statistical Analysis**

Data were tabulated using Microsoft Excel and analyzed using SPSS software (v 21.0; SPSS Inc., Chicago, IL). Baseline data were presented using mean ± standard deviation or frequency (%). Continuous variables were analyzed using unpaired student’s t-test. P-values less than 0.05 were considered significant.

## Results

**Baseline characteristics of participants**

**Figure 1:**
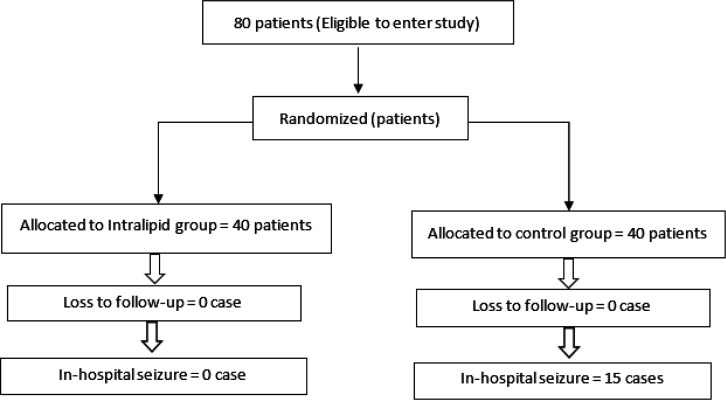
Study enrollment and distribution of studied patients

80 patients who abused tramadol and met the study criteria were randomly assigned to intervention (40 cases) or control (40 cases) groups. Figure 1 demonstrates the breakdown of all patients enrolled in the study. Seizure had occurred in 44 (56%) patients before admission to the emergency department. Table 1 compares the baseline characteristics of patients between the groups. There were not any statistical differences between the groups regarding sex distribution (p = 0.513) and mean age (p = 0.19), presenting vital signs (p < 0.05), laboratory findings (p < 0.05), and mean abused dose of tramadol (p = 0.472) as well as occurrence of prehospital seizure (p = 0. 7).

**Outcomes**

Table 2 compares the studied outcomes between the two groups. In-hospital seizure occurred in 15 (18.75%) cases (all in control group; p < 0.001). All documented in-hospital seizures were tonic-colonic. The mean duration of admission was 2.01 ± 1.13 days in the control group and 2.15 ± 1.04 days in the intervention group (p = 0.6). The number needed to treat for ILE to prevent tramadol-induced seizure was 2.7 (37.5% absolute risk reduction). It means that 2.7 patients should be treated with ILE so that it can prevent one tramadol-induced seizure. 

## Discussion

Current findings advocate ILE administration, as an adjunct to standard antidote protocols, in tramadol intoxication to prevent tramadol-induced seizures. 

ILE has been approved for parenteral nutrition and as a vehicle for lipophilic medicines by the Food and Drug Administration (FDA) since a long time ago. However, several studies have reported the efficacy of using ILE as an antidote in poisoning with different drugs with no reported adverse effects. The first clinical study conducted by Rosenblatt in 2006 showed that ILE infusion attenuates CNS toxicity of local anesthetics, and increases consciousness score on Glasgow coma scale (9).

The use of opioids, including tramadol, and subsequent poisoning have overwhelmingly increased in recent years (10). The most common symptoms of tramadol intoxication are a seizure, various cardiac dysrhythmias, and in some cases death. Seizure is the most important presentation of tramadol overdose, and it has been reported in 54.4% of the patients (11, 12).

A recent experiment showed the efficacy of ILE administration in managing tramadol poisoning (8). In the current research, ILE was able to attenuate tramadol-induced seizures, and was efficacious up to 100% with 12 mL/kg doses.

The occurrence of seizures after tramadol consumption has been reported in many studies. Several studies noted the incidence of seizures after tramadol overdose, while some other studies claimed the dose-independent manner of this phenomenon (13). According to the results, 44 out of 80 patients (56.2%) admitted to the emergency department had experienced a seizure before entering the emergency department. In the current study, 15 patients (37%) in the control group experienced a seizure during the hospital admission period. Talaei et al. described seizures in 46% of tramadol abusers (13),  while Jovanović-Čupićnoted reported that 54.4% of patients experienced seizure with the dose range of 250–2500 mg (14).

In this study, the lowest dose resulting in seizure was 50 mg, which was in contrast with results by Marquardt et al. (15), Spiller (16), and Farzaneh et al. (17), who reported the lowest dose of tramadol leading to seizure as 200, 500, and 1000, respectively. In a case report by Beyaz et al. 75 mg of tramadol caused a tonic-colonic seizure (18). Taghaddosinejad and colleagues (19) reported two cases of seizure at a dose of 100 mg and the results of our study is in accordance with these findings.

This study was built upon the previous study by Vahabzadeh et al., which focused on the effect of intralipid infusion on seizure prevention in rabbits with acute tramadol intoxication. They demonstrated the positive effect of ILE on seizure prevention, which is consistent with the findings of the present study (20).

According to the results, ILE can be used as an effective antidote against seizure and possibly other adverse outcomes in patients with tramadol intoxication. Unfortunately, there is no high-quality controlled clinical study in this regard and we suggest performing large-scale controlled clinical trials to assess lipid emulsion therapy as a first-line therapy for indications such as tramadol poisoning. Lipid emulsions may have some adverse side effects. We suggest performing larger prospective studies along with patient follow-up in the future to elucidate the possible therapeutic effects and latent complications of ILE in tramadol-induced toxicities.

**Table 1:** Comparison of baseline characterises of patients between the groups

**Parameters**	**Intervention (n = 40)**	**Control (n = 40)**	**P-value**
**Sex **			
Male	34 (85)	36 (90)	0.513
Female	6 (15)	4 (10)	
**Age (year)**			
Mean ± SD	25.1 ± 8.1	22.8 ± 5.7	0.190
**Vital sign (on admission)**			
Glasgow coma scale	13.65 ± 1.76	13.63 ± 1.87	0.951
Systolic blood pressure(mmHg)	118.5 ± 8.7	121.7 ± 1	0.177
Diastolic blood pressure(mmHg)	77.5 ± 9.6	77.3± 10.2	0.842
Heart rate (bpm)	85.7 ± 8	84.3 ± 7.5	0.463
Respiratory rate /minute)	15.2 ± 3	14.5 ± 4.2	0.547
SaO_2_ (%)	94.0 ± 2.5	93.0 ± 3.4	0.587
**Laboratory findings**			
Tramadol dose(mg)	229.8 ± 135.5	195 ± 156.5	0.472
Blood Sugar (mg/dL)	112 ± 44.6	109 ± 39.5	0.682
Serum sodium (mEq/L)	138.3 ± 3.1	137.8 ± 3.2	0.520
Serum potassium (mEq/L)	4.3 ± 0.36	4.2 ± 0.39	0.119
Creatinine (mq/dL)	0.91 0.16	0.89 ± 0.15	0.791
pH	7.39 ± 0.37	7.39 ± 0.36	0.830
ALT (IU/L)	24.2±10.3	27±9.5	0.648
AST(IU/L)	34.92±9.31	35.07±13.1	0.195
BUN(mg/dL)	25.4 ± 7.3	26.1 ± 6.6	0.547
Hemoglobin (g/dL)	13.8± 1.5	13.6± 1.8	0.842
**Pre-hospital seizure **			
Yes	21 (52.5)	23 (57.5)	0.700
No	19 (47.5)	17(42.5)	

Data are presented as mean ± standard deviation (SD) or frequency (%). ALT: alanine aminotransferase; AST: aspartate aminotransferase; BUN: blood urea nitrogen.

**Table 2:** Comparison of studied outcomes between the groups

**Outcome**	**Intervention (n = 40)**	**Control (n = 40)**	P-value
**In-hospital seizure**			
Yes	0 (0.00)	15 (37.5)	<0.001
No	40(100)	25(62.5)	
**Duration of hospitalization (Days)**			
1	14 (35.0)	18 (45.0)	0.616
2	12 (30.0)	9 (22.5)	
3	11 (27.5)	10 (25.0)	
4	2(5.0)	1(2.5)	
5	1(2.5)	2(5.0)	

Data are persecuted as number (%).

## Limitations

There were some limitations to our research. The study was done as a pilot with relatively limited participants. In this respect, another multicenter analysis must be done with a greater sample size. Considering the incomplete information about the history of using tramadol or other drugs in patients, the analysis, and comparison between the history tramadol use and seizure was not possible.

## Conclusion:

The findings of this study supported ILE administration, as an adjunct to standard antidote protocols, in tramadol intoxication to prevent tramadol-induced seizures. However, larger prospective studies along with patient follow-up should be performed in the future to elucidate the possible therapeutic effects and latent complications of ILE in tramadol-induced toxicities.
